# Evaluation of a clinical decision support system for dermatology in a remote area: insights from Martinique

**DOI:** 10.3389/fmed.2025.1555803

**Published:** 2025-10-30

**Authors:** Alice Callens, Moustapha Drame, Julia Dugardin, Emilie Baubion, Adrien Perrier, Romain Blaizot, Nicola Briand, Emmanuelle Amazan, Gladys Ferrati-Fidelin, Ferdaous Khammouma, Nicolas Olivier, Melissa Heleine, Aziz Baccouche, Raymond Helenon, Aurélie Martin, Claire Jacquin, Anne-Laure Messagier

**Affiliations:** ^1^Dermatology Department, CHU Pierre Zobda-Quitman, Fort-de-France, Martinique; ^2^Epidemiology, Health Economics Department, CHU Pierre Zobda-Quitman, Fort-de-France, Martinique; ^3^Diabétology and Endocrinology, CHU Pierre Zobda-Quitman, Fort-de-France, Martinique; ^4^Dermatology Departement, CHU de Guyane, Cayenne, French Guiana; ^5^Private Dermatology Practice, Fort-de-France, Martinique; ^6^Infectiology Department, CHU, Nîmes, France; ^7^Private Dermatology Practice, Nouméa, New Caledonia

**Keywords:** dermatology, general medicine, clinical decision support system, digital tool, e-health, dermagic

## Abstract

**Background:**

Dermatology faces workforce shortages in Martinique, a French Overseas Department, where general practitioners are often the first point of care. The lack of dermatology CDSS tools adapted to tropical and Francophone contexts, especially across diverse phototypes, underscores the need for innovative digital solutions.

**Objective:**

The Research Objective (RO) of this study was to evaluate a newly developed dermatology CDSS (“Dermagic”) in Martinique, focusing on its reliability, usefulness, and effectiveness across diverse phototypes. To guide this evaluation, we formulated three Research Questions (RQ): What evidence supports acceptance and relevance among physicians? Which barriers and underused features may guide future improvements? How adaptable is the tool to other Francophone or tropical regions?

**Methods:**

A cross-sectional study was conducted from February 7 to March 7, 2024, using a 21-item questionnaire aligned with HONcode and Netscoring criteria. The survey was sent to 117 physicians; 64 responses were analyzed. Reliability, usefulness, and satisfaction were assessed, including the System Usability Scale (SUS). Subgroup analyses were performed using exact Mid-P tests to explore variability of use across specialties.

**Results:**

Respondents (response rate 55%) highlighted the tool’s quick accessibility (98.4%), improved prescription facilitation (100%), ergonomic design (90.6%), and adaptability to local dermatology needs, including diverse phototypes (95.3%). Regular use was reported by 62% of physicians, with 20% using the tool daily and 42% weekly. Subgroup analysis revealed higher use of the Medical Coding module among dermatologists compared to other non-generalist specialists (*p* = 0.016), while no significant differences were observed between dermatologists and general practitioners. A high System Usability Scale (SUS) score of 87.7/100 indicated excellent usability, and 96% of users reported being satisfied.

**Conclusion:**

This CDSS is a reliable, user-friendly tool that supports dermatological practice in Martinique, particularly addressing the management of diverse phototypes and the needs of local doctors and patients. Limitations include the modest sample size and self-reported nature of responses. Future directions include cross-regional evaluations, integration of AI for diagnostic support, and independent validation studies to strengthen its scalability and impact.

## 1 Introduction

General practitioners frequently address dermatological issues. In France in 2022, 6.2% of general practice consultations involved skin conditions (1). While a third of French patients consult a general practitioner first for dermatoses, as recommended by the coordinated care pathway (2), the average waiting time for a dermatologist is 61 days compared to 6 days for a general practitioner (3). These delays are even more pronounced in Martinique, where only 13 dermatologists serve the population, representing 3.73 practitioners per 100,000 inhabitants, significantly below the national average (4). The supply of dermatological care continues to decline (5), and access to dermatological care remains difficult in Martinique.

Martinique’s unique demographic, predominantly of African descent with darker skin phototypes (IV, V, VI), presents specific challenges in diagnosing and managing dermatoses (6, 7). Historically, dermatological research and resources have centered on Caucasian skin, leaving practitioners less familiar with the presentation of conditions on darker phototypes (8).

The average consultation with a general practitioner in France lasts 16 min (9). Practitioners therefore need to be fast and efficient in responding to their patients’ needs within this limited timeframe, while guaranteeing optimal care. Clinical Decision Support Systems (CDSS) can help save consultation time (10), particularly by streamlining information retrieval, thereby allowing more time for informing patients, answering their questions, and providing therapeutic education.

**Figure 1:**
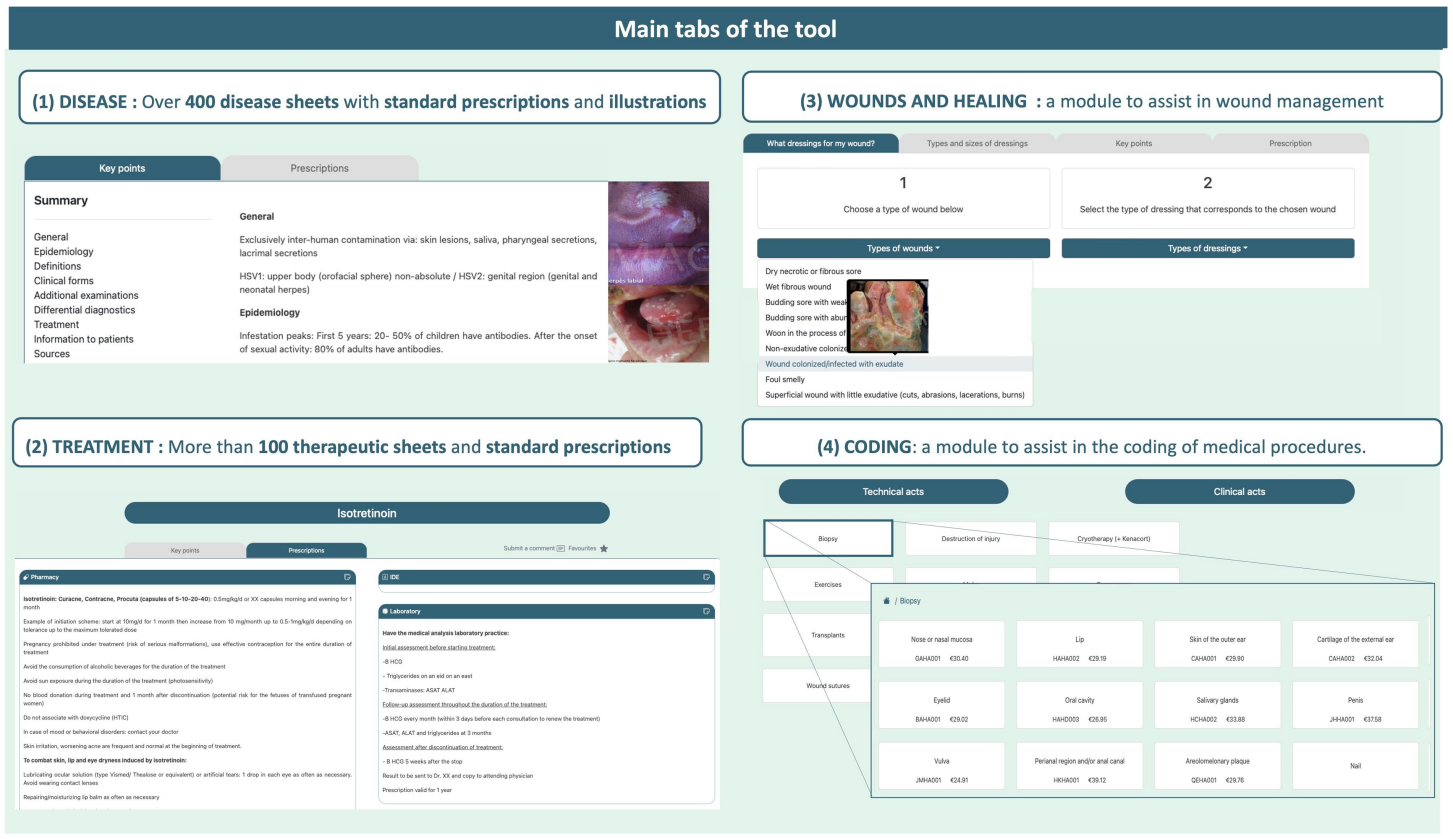
Tool’s overview. The figure shows the four main modules: diagnostic guide (Diseases tab), therapeutic options (Treatments tab), wound care (Wounds and Healing tab), and medical coding (Coding tab).

Clinical Decision Support Systems (CDSS) have emerged as valuable tools to assist physicians by providing timely, filtered, and relevant clinical information, improving decision-making and patient care (11, 12). With the growing importance of digital tools in healthcare, this study evaluates a newly developed dermatology CDSS (“Dermagic”), designed for use on computers or smartphones with internet access. This tool is organized into four distinct modules (Figure 1):

- Diagnostic Guide (Diseases tab), which includes over 400 summarized skin conditions and treatments with a photo library adapted to various phototypes.
- Therapeutic Options (Treatments tab), summarizing more than 100 commonly used therapies in dermatology.
- Wound Care (Wounds and Healing tab), providing dressing guides.
- Medical Coding (Coding tab), offering support for billing procedures.

The Research Objective (RO) of this study is to evaluate this dermatology CDSS, in Martinique, with a focus on its reliability, usefulness, and effectiveness in supporting practitioners across diverse skin phototypes. To guide this evaluation, we formulated the following Research Questions (RQ):

- What evidence supports the acceptance and perceived relevance of the tool among physicians in Martinique? (RQ1).
- Which barriers and underused features may guide future improvements? (RQ2).
- How adaptable is the tool to other Francophone or tropical regions? (RQ3).

## 2 Materials and methods

### 2.1 General principles

A cross-sectional descriptive study was conducted. The questionnaire was emailed to 117 doctors on February 7, 2024, and responses were collected until March 7, 2024. Inclusion criteria required participants to have used the tool and practiced in Martinique between May 2023 (tool creation) and March 2024. Exclusion criteria included doctors who had never used the tool and/or never practiced in Martinique. Four participants were excluded due to incompatible practice locations. A total of 64 questionnaires were analyzed.

### 2.2 Choice of quality assessment framework

Several tools exist for evaluating healthcare websites, but none are currently validated by French health authorities. Therefore, the questionnaire was designed using the charter from the University Department of General Medicine (DUMG) at Paris Diderot (created in 2014). This charter, recognized as a benchmark in healthcare internet media research, combines criteria from HONcode and Netscoring, previously validated by the French National Authority for Health (HAS). The framework has been referenced in multiple publications and is widely used for new CDSS evaluations.

### 2.3 Questionnaire design

The questionnaire consisted of 21 straightforward questions. Participants were contacted by email, responses were anonymized prior to analysis. Participation was voluntary and unpaid. In accordance with the tool’s Terms of Use, physicians agreed at registration that they could be contacted by email for evaluation surveys, satisfaction questionnaires, or academic research (including theses and scientific articles), which constituted prior consent to receive this questionnaire. Given the absence of health-related data collection and in line with French regulations, ethics board approval was not required for this study. The questionnaire featured a mix of multiple-choice and open-ended questions. To evaluate user satisfaction, the System Usability Scale (SUS) was included, comprising 10 questions on a Likert scale, producing a score out of 100. Scores were classified as follows:

- Poor: 0–51/100.
- Acceptable: 52–75/100.
- Good: 76–85/100.
- Excellent: 86–100/100.

### 2.4 Definition of quality criteria

The tool’s evaluation focused on reliability, usefulness, and satisfaction, which were detailed in a structured framework (Table 1).

**Table 1:** Quality criteria.

Category	Subcategory	Criteria	Examples
Reliability	Context	Objectives	Clear announcement of site objectives
			Identifying the authors
		Independence	Site financing declaration
			No advertising
			No conflict of interest
	Contents	Accuracy	Clearly identified and documented information sources
			Quoting original sources
		Update	Regular content updates in line with the latest recommendations
			Update date visible on every page of the site
	Interface	Accessibility	Free registration
			Intuitive site name
		Ergonomics	Logical site organization
			Easy navigation
			Fast page loading
			Easy-to-read text and images
			Sober design
		Interactivity	Write comments and ask questions
		Security	Protection of user data in accordance with confidentiality standards
Useful	Contents	Resources	Easy access to information
			Easy access to dermatology recommendations
	Context	Special features	A tool adapted to the specific characteristics of Martinique
		Diversity	Access to illustrations on any type of phototype
	Benefits	In consultation	Time saving
			Fewer visits to dermatologists and other specialists
		For the doctor	Knowledge enhancement
			Builds confidence in patient care
Satisfaction	Users’ opinions	Experience	Overall user satisfaction
		Recommendations	Recommend the tool to a colleague
		Comparison	Improvements over existing tools

### 2.5 Data analysis

All collected data were anonymized and analyzed using descriptive statistics. A socio-demographic profile of respondents was compared with official national benchmarks. Chi-square tests were used to assess differences in distributions for gender, age, and medical specialty (see Results, Table 2).

**Table 2:** Comparative socio-demographic profile of study respondents and official medical benchmarks (13, 14).

Characteristic	Number (*n* = 64)	Percentage (%)	Official benchmark ^(b)^	Δ (% points)	χ^2^ (*p*-value)
Gender					18.06 (*p* < 0.001)
Male	15	23.4	50% (France, 2023)	−26.0	
Female	49	76.6	50 (France, 2023)	+26.0	
Age					65.54 (*p* < 0.001)
25–39 years old	50	78.1	31% (<40 y, France, 2023)	+47.1	
40–49 years	4	6.3	16% (France, 2023)	−9.7	
>50 years	10	15.6	53% (≥50 y, France, 2023)	−37.4	
Medical specialty					498.24 (*p* < 0.001)
General medicine	29	45.3	45.8% (Martinique, INSEE 2023)	−0.5	
Dermatology	23	35.9	1.5% (France, 2023)	+34.4	
Other ^(a)^	12	18.8	53% (France, 2023)	−34.2	
Number of years			Not available	Not available	Not available
<5 years	16	25.0			
5–9 years	25	39.1			
10–19 years	11	17.2			
>20 years	12	18.8			

(a) Nephrology, internal medicine, rheumatology, geriatrics, internal medicine, hematology. (b) National DREES 2023 data used for sex and age distribution (approximate categories: <40, 40–49, ≥50 years). Detailed regional data for dermatology in Martinique are not always publicly available; national DREES 2023 data were used as an approximation.

In addition, a complementary subgroup analysis was conducted concerning the use of the Wound Care and Medical Coding modules. For these analyses, responses regarding the completeness of each section were dichotomized into two categories (see Results, Table 3):

- Likely users: respondents who answered “Yes, definitely», «Rather yes,” “Rather no,” “Not at all” (i.e., respondents who had viewed the section, regardless their evaluation).
- Unlikely users: respondents who answered, “I did not use it.”

**Table 3:** Content quality assessment by module (user ratings in %).

Category	Response option	Is the “Wound Care” section sufficiently complete?	Is the “Medical Coding” section sufficiently complete?
Likely users	Yes, definitely	21.9	25.0
	Rather yes	42.2	17.1
	Rather no	3.1	1.6
	Not at all	0	0
Unlikely users	I did not use it	32.8	56.3

These categories were used to perform subgroup comparisons between specialties with exact Mid-P tests, using OpenEpi software. The significance threshold was set at 5%. This approach was chosen for its accuracy in small sample sizes (see Results, Table 4).

**Table 4:** Subgroup comparisons of module use by specialty.

Module	Comparison	Likely users	Unlikely users	*p*-value*
Medical coding	Dermatologists / Other specialties	14/2	9/10	0.016
	Dermatologists / General practitioners	14/12	9/17	0.178
	General practitioners / Other specialties	12/2	17/10	0.147
Wound care	Dermatologists / Other specialties	14/6	9/6	0.561
	Dermatologists / General practitioners	14/22	9/7	0.266
	General practitioners / Other specialties	22/6	7/6	0.131

*p < 0.05 considered statistically significant.

For the evaluation of reliability, usefulness, and satisfaction, categorical responses were recoded into ordinal numerical values to allow calculation of mean scores:

- Dichotomous items: “Yes” = 5, “No” = 1, and “Do not know” = 3;
- Likert-type item: the 4-point scale was recoded as 5 (“very satisfied”), 4 (“somewhat satisfied”), 2 (“somewhat dissatisfied”), and 1 (“not at all satisfied”).

Item scores were then averaged within each predefined domain to obtain composite domain scores (see Results, Table 5).

**Table 5:** Aggregated domain scores.

Domain	Number of items	Mean score (1–5)
Usefulness	9	4.74
Reliability	10	4.42
Satisfaction	3	4.76

## 3 Results

### 3.1 Most respondents were young physicians and primarily general practitioners

Table 2 summarizes the diversity of respondents in terms of age, gender, and career stage, providing useful context for exploring variability of use and potential scalability (QR3). Among responding physicians, the majority were women (77%) and most were early in their career, aged 25–39 years (78%) with less than 10 years of practice (64%). Almost one-fifth, however, had more than 20 years of experience (19%), reflecting adoption across different stages of practice. General practitioners formed the largest group (45%), followed by dermatologists (36%) and other specialists (19%).

Compared with official benchmarks (13, 14), our sample included more women and younger physicians, while the proportion of general practitioners was close to the regional distribution and dermatologists were markedly over-represented. These differences were statistically significant for gender, age, and specialty (all *p* < 0.001).

### 3.2 Physicians rarely used CDSS when seeking dermatology information

According to Table 6, physicians’ information-seeking habits highlight the limited baseline use of CDSS and emphasize the relevance of evaluating such a tool (QR1). When uncertain about a skin condition, physicians mainly used official websites and general search engines, while about one-third consulted books or atlases. Fewer (20%) relied on CDSS, most often dermatology-specific tools (30%).

**Table 6:** Looking for information about a skin problem.

Item	Number (*n* = 64)	Percentage (%)
Information sources used		
Dermatology book or atlas	22	34.4
Official websites (e.g: French Society of Dermatology, French Health Authority…)	46	71.9
General search engines	28	43.8
Other (CDSS, tele-expertise or colleague’s opinion)	13	20.3
CDSS* used in dermatology		
Specific dermatology tool (e.g: Therapeutics in Dermatology…)	19	29.7
General medical tool (e.g: Vidal Recommendations…)	2	3.1
None	4	10.8
No answer	12	32.4

* CDSS: Clinical Decision Support System.

### 3.3 Peer recommendations were the main source of tool awareness

The predominance of peer-to-peer recommendation reflects early signs of professional acceptance and diffusion of the tool (QR1). Most respondents learned about the tool through colleagues (70%), while a dermatology training day organized for private practitioners in Martinique accounted for most of the remaining introductions (23%).

### 3.4 Most physicians used the tool regularly

Patterns of frequency and duration of use provide indirect evidence of physicians’ acceptance of the tool in their daily practice (QR1). Regular use was reported by 62% of physicians, including weekly use (42%) and daily use (20%). Almost two-thirds had used the tool for at least 3 months.

### 3.5 Relevance of the tool

#### 3.5.1 Diagnostic and therapeutic modules were widely used

As detailed in Tables 3 and 4, patterns of use across the different modules reveal potential barriers and underused features (QR2). The tool’s four main modules (‘Diagnostic Guide’, ‘Therapeutic Options’, ‘Wound Care’, and ‘Medical Coding’) are summarized in Figure 1. Most users found the Diagnostic Guide and Therapeutic Options comprehensive (89 and 88%), reflecting strong uptake of these modules. In contrast, the Wound Care module was less frequently used despite 64% finding it comprehensive. The Medical Coding module was the least accessed, with 56% of users not consulting it and only 42% finding it sufficiently detailed.

To better understand the limited use of certain modules, a subgroup analysis was conducted to explore whether usage patterns varied by medical specialty (dermatology, general medicine, other specialties). Differences in module use between specialties offer insight into variability among users and implications for scalability (QR3). Dermatologists were significantly more likely to use the Medical Coding module compared to physicians from other specialties (*p* = 0.016). However, no statistically significant differences were observed between general practitioners and dermatologists (*p* = 0.178), nor between general practitioners and other specialists (*p* = 0.147). Similarly, usage of the Wound Care module did not differ significantly across specialties (*p* > 0.05).

#### 3.5.2 Physicians considered the tool reliable

According to Table 7, findings on transparency of objectives, absence of conflicts of interest, and data protection provide insight into physicians’ perceptions of reliability (QR1). Most respondents (≥ 80%) considered the site reliable, citing clear goal announcements, absence of conflicts of interest or advertising, and well-documented sources. A large majority (≥80%) also found the recommendations up to date and appreciated the site’s ergonomics. In contrast, data protection was judged adequate by just over half of respondents (58%), while the possibility of interacting with authors was poorly recognized, reported by only a quarter (25%) and largely unknown to others (61%).

**Table 7:** Reliability.

Item	Number (*n* = 64)	Percentage (%)
The announcement of the site’s objectives is clear		
Yes	59	92.2
No	1	1.6
Do not know	4	6.3
No conflicts of interest are evident on the site		
Yes	53	82.8
No	2	3.1
Do not know	9	14.1
No advertising on the site		
Yes	59	92.2
No	2	3.1
Do not know	3	4.7
Information sources are easily identifiable and clearly documented		
Yes	54	84.4
No	0	0.0
Do not know	10	15.6
The recommendations relating to pathologies and treatments are updated and the update dates can be viewed on the website.		
Yes	51	79.7
No	0	0.0
Do not know	13	20.3
User data is protected in accordance with confidentiality standards		
Yes	37	57.8
No	1	1.6
Do not know	26	40.6
The site provides a description of the financing and its sources of income		
Yes	30	46.9
No	2	3.1
Do not know	32	50.0
Ergonomics are satisfactory (design, navigation, organization, page loading, readability).		
Yes	58	90.6
No	5	7.8
Do not know	1	1.6
Opportunity to interact on the site (questions, comments, remarks)		
Yes	16	25.0
No	9	14.1
Do not know	39	60.9
Registration is free		
Yes	59	92.2
No	0	0.0
Do not know	5	7.8

#### 3.5.3 The tool improved prescribing, confidence, and saved time in consultations

As presented in Table 8, the reported benefits in terms of rapid access to recommendations, easier prescribing, and improved confidence directly reflect the perceived utility and relevance of the tool (QR1). Almost all physicians reported quick access to recommendations (98%) and easier prescribing (100%). The tool was considered well adapted to local practice (95%), enhanced dermatology knowledge (97%), and improved confidence in patient management (91%). Many also noting a reduced need to consult a dermatologist for straightforward cases (84%). In addition, a large majority reported time savings during consultations (88%).

**Table 8:** Usefulness.

Item	Number (*n* = 64)	Percentage (%)
It provides easy access to additional resources		
Yes	57	89.1
No	1	1.6
Do not know	6	9.4
It provides quick and practical access to recommendations in dermatology		
Yes	63	98.4
No	1	1.6
Do not know	0	0.0
It facilitates the prescription of dermatological treatments		
Yes	64	100.0
No	0	0.0
Do not know	0	0.0
It is adapted to the practice of dermatology in Martinique		
Yes	61	95.3
No	0	0.0
Do not know	3	4.7
It provides access to illustrations on all types of phototypes		
Yes	52	81.3
No	7	10.9
Do not know	5	7.8
It boosts physician confidence in the management of dermatological problems		
Yes	58	90.6
No	4	6.3
Do not know	2	3.1
It enhances the physician’s knowledge of dermatology		
Yes	62	96.9
No	0	0.0
Do not know	2	3.1
Its use saves consultation time		
Yes	56	87.5
No	6	9.4
Do not know	2	3.1
It allows you to seek less advice from a dermatologist or other specialist		
Yes	54	84.3
No	6	9.4
Do not know	4	6.3

#### 3.5.4 Physicians reported high satisfaction and usability scores

Table 9 shows high SUS scores and strong satisfaction rates, reflecting broad acceptance of the tool among physicians (QR1). The tool achieved an excellent usability score (87.7/100) on the System Usability Scale. Overall, almost all users were satisfied (96%), including a large majority who were very satisfied (73%). Nearly 9 in 10 would recommend the tool to colleagues, and more than 4 in 5 believed it provided improvements over existing CDSSs (84%).

**Table 9:** User satisfaction.

Item	Number (*n* = 64)	Percentage (%)
User experience		
Very satisfied	47	72.5
Somewhat satisfied	15	23.5
Somewhat dissatisfied	2	3.9
Not at all satisfied	0	0.0
Recommend the site to colleagues		
Yes, that’s right	57	89.1
Rather yes	5	7.8
Rather no	2	3.1
Not at all	0	0.0
Significant improvement over existing tools		
Yes, that’s right.	52	83.9
Rather yes	9	14.5
Rather no	1	1.6
Not at all	0	0.0

#### 3.5.5 Overall evaluation shows strong acceptance of the tool

Table 5 summarizes the integrated overview, with categorical responses from Tables 7–9 aggregated into three domains. The synthesis highlights strong overall acceptance of the tool. Usefulness (4.74/5) and satisfaction (4.76/5) were very high, while reliability was positive though slightly lower (4.42/5).

## 4 Discussion

### 4.1 Respondent profile reflects local recruitment

The tool was designed for use by all practitioners, regardless of their specialty. In this study, the sample included 29 general practitioners (45%), 23 dermatologists (36%), and 12 doctors from various specialties (19%), ensuring representation of different specialties, although the distribution did not fully match official benchmarks. Compared with official benchmarks, the proportion of general practitioners was close to the regional distribution, while dermatologists were strongly over-represented. Female physicians accounted for 76.6% of respondents, which is consistent with the increasing feminization of the medical profession in France (15) but remains higher than the 50% reported in national statistics. These imbalances can be explained by the recruitment context: the tool was created and first disseminated within the dermatology department of Martinique, which led to a high proportion of dermatologists among respondents. As almost all dermatologists on the island are women, this also contributed to the over-representation of female physicians in our sample. Most respondents were early in their career, aged 25–39 years (78%), whereas national data indicate that only about 31% of physicians are under 40 (13, 14). This over-representation of younger physicians likely reflects both the local context, where many positions are filled by residents and young doctors due to frequent turnover, and their greater propensity to adopt digital health solutions. Younger doctors are generally more likely to use Clinical Decision Support Systems (CDSSs) (16), yet it is noteworthy that 19% of respondents had more than 20 years of experience, showing that the tool was also adopted across different stages of practice. This diversity of respondents provides valuable context for interpreting variability of use and scalability (QR3).

### 4.2 Decision support systems (CDSS) use remains limited despite known benefits

The abundance of information sources in medicine and the constant updating of recommendations often make it difficult for doctors to make decisions. In our study, practitioners searched for information primarily on official websites (71.9%), on general search engines (43.8%), and only 20% used CDSS. According to the general medicine department of Paris-Diderot, the use of CDSS is perfectly compatible with an Evidence Based Medicine (EBM) approach (17). A systematic review of the international literature has presented the various obstacles preventing the integration of CDSS in primary care (18). The main obstacle to their use is said to be technical (computer problems during use, lack of integration with business software, lack of training…). However, the Dutch review by Niazkhani (19) evaluating the impact of CDSS on the work process, showed that in addition to improving the quality and safety of care, these tools increase caregivers’ productivity and facilitate their work. They reduce prescription errors and improve adherence to recommendations. These results situate our findings within physicians’ information-seeking behaviors and underscore both the opportunities and the challenges associated with CDSS adoption in routine practice (QR2).

### 4.3 The tool is reliable, independent, and accessible

The evaluated tool is built on validated and recognized scientific resources, including publications from French and international dermatology societies. Most users (84%) found the sources easily identifiable, with bibliographic references accessible directly from the platform. Notably, 92% of users appreciated the absence of advertising and conflicts of interest, reinforcing the tool’s independence from commercial influence. Furthermore, compliance with General Data Protection Regulation (GDPR) ensures robust data protection (20), although only 57.8% of users explicitly recognized this feature. Offering the tool free of charge is another key factor in ensuring accessibility to a wide range of practitioners, particularly in low-income countries where financial constraints might otherwise limit access to such resources.

In comparison with “Dermatoclic,” another French CDSS evaluated among 26 general practitioners and covering around 120 dermatological conditions without an integrated photo library (21), our tool covers more than 400 skin diseases and includes an iconography adapted to different phototypes. Moreover, while “Dermatoclic” is mainly intended for general practitioners, our tool targets a broader audience, including not only General practitioners but also dermatology residents, dermatologists, and other specialists involved in skin disease management. These differences strengthen the relevance of its evaluation and highlight the need for future comparative studies. Similarly, international CDSS such as “VisualDx” illustrate another model, with a subscription-based system covering around 3,000 conditions and more than 40,000 clinical images, validated by expert editorial boards (22). While powerful and widely adopted by institutions the cost and need for institutional licensing may limit accessibility for some practitioners, in contrast to our tool, which is freely available. This comparison, when framed within our overall research objective, positions our tool as a scalable and accessible solution for under-resourced settings, emphasizing both its reliability and potential adaptability (QR1, QR3).

### 4.4 The tool is perceived as useful but some modules remain underused

The simplicity of the tool was highlighted by 89.1% of users, who reported streamlined access to comprehensive resources, particularly in the “Diagnostic Guide” (94%) and “Therapeutic Options” (87%) sections. These features contributed to improved confidence in patient management (90.6%) and dermatological knowledge (96.9%). However, the “Wound Care” and “Medical Coding” tabs were less frequently consulted. The limited uptake of the “Wound Care” and “Medical Coding” modules illustrates barriers to adoption and points to areas for interface and training improvements (QR2). This may be partly explained by their limited visibility, as they do not appear directly on the homepage. Future improvements could include better highlighting these sections and providing short educational videos to guide users through the platform’s full range of functionalities. Subgroup analysis suggested a higher use of the Medical Coding module among dermatologists compared with other specialists. This is consistent with the fact that dermatologists are more frequently confronted with coding requirements for dermatological procedures, whereas wound care is a cross-cutting skill shared by different specialties. However, as no other significant differences were observed, this isolated finding should be interpreted with caution, and factors beyond medical specialty are likely to influence engagement with these modules.

Additionally, 87.5% of users reported time savings during consultations, a critical benefit in clinical practice. Regular use of the tool (62%) underscores its practicality, with many users visiting the site weekly or even daily. These patterns of regular use and reported savings further support the tool’s perceived utility and relevance in daily practice.

### 4.5 Strong suitability for Martinique’s population and clinical context

**Figure 2:**
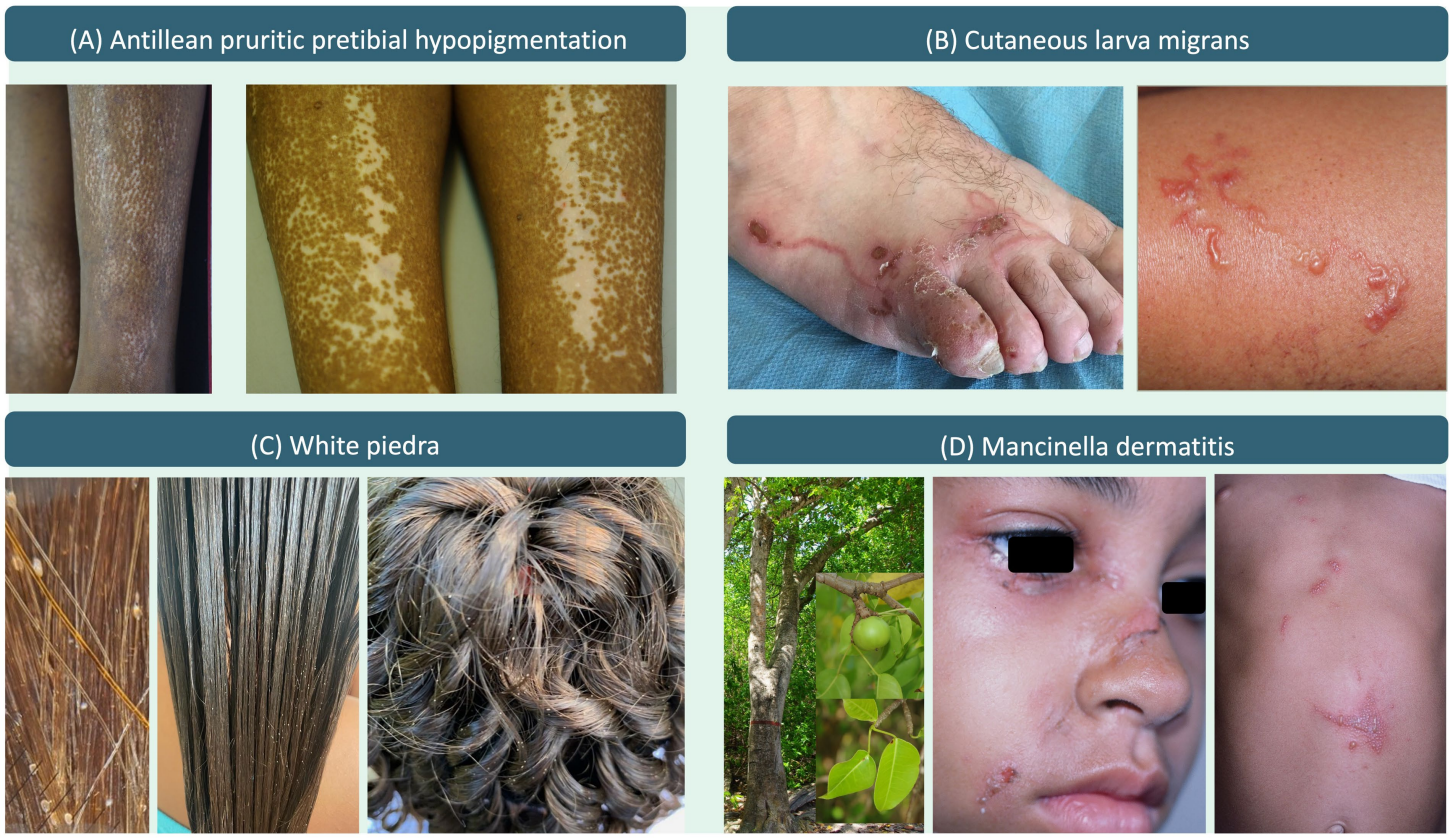
Tool’s examples of dermatoses observed in Martinique: **(A)** Antillean pruritic pretibial hypopigmentation, **(B)** Cutaneous larva migrans, **(C)** White piedra (clinical and dermoscopy views), **(D)** Mancinella dermatitis.

Martinique’s unique population, predominantly composed of individuals with dark skin of African descent (95%), presents distinct challenges in diagnosing dermatoses due to variations in presentation across phototypes (6). In our study, 95% of users confirmed the tool’s suitability for local practice, with 81% emphasizing the value of its diverse iconography. This highlights the need for tools tailored to specific demographic and environmental contexts, such as the tropical climate and genetic diversity of Martinique [(23–26) and Figure 2]. These findings provide tentative insights into user variability (QR3) and may indicate the need to adapt certain functionalities to different professional profiles. An evaluation of the tool in other territories, notably in other French overseas departments, mainland France, and broader Francophone or tropical regions worldwide, should be considered in the future.

Beyond Martinique, the tool could be generalized to other French overseas departments (e.g., Guadeloupe, French Guiana, Réunion, Mayotte) and to other Francophone or tropical regions where epidemiological profiles and healthcare organization show similarities. Scaling up digital health solutions relies on shared principles such as adaptability, interoperability, infrastructure compatibility, user-friendliness, and stakeholder engagement (27, 28). Our tool meets these criteria through its modular design (‘Diagnostic Guide’ with disease summaries and a photo library covering different phototypes, ‘Therapeutic Options’ with therapeutic sheets, ‘Wound Care’ with dressing guides, and ‘Medical Coding’ for billing support), its intuitive interface, and its user-centered development, in line with best practices described by international analyses. However, the applicability of therapeutic sheets will need to be adapted to local contexts, as some treatments available in Francophone regions may not be accessible in other resource-limited countries. Nevertheless, as highlighted in another analysis of digital health deployment, several barriers remain, including heterogeneous infrastructures, diverse political and regulatory environments, and the need for independent evaluations to confirm the reproducibility of results in different settings (29). Therefore, we acknowledge the need for additional evaluations in other Francophone regions to confirm the usability and impact of the tool. These considerations underline how local adaptation is essential to inform broader scalability in other Francophone or tropical regions (QR3).

### 4.6 High satisfaction confirms the tool’s acceptance in daily practice

The tool achieved an excellent usability score of 87.7/100 on the System Usability Scale (SUS). Satisfaction levels were high, with 96% of users expressing contentment, including 72.5% who were very satisfied. Furthermore, nearly 97% of users said they would recommend the site to one of their colleagues, which also underlines their satisfaction with the tool. In addition, 62% of respondents reported using the tool on a regular basis (weekly or daily), demonstrating its integration into routine clinical activity. A large majority (98.4%) believed that the tool offers significant improvements over existing tools, which suggests that this new CDSS will be able to play a tangible role for doctors in doctors’ dermatological practice. These results further illustrate the acceptance and relevance of the tool in daily practice (QR1).

### 4.7 Limitations: impact on representativeness and generalizability

The response rate to the feedback survey was 55% (64/117), which, although modest, is comparable to the rates usually reported in physician surveys, often ranging between 30 and 40% (30, 31). Nevertheless, a non-response bias cannot be excluded, and our sample may not fully represent all physicians in Martinique. In particular, a fully representative evaluation would have required access to the complete list of physicians practicing in Martinique during the study and their actual use of the tool. In practice, such data are not available, as physicians frequently change their practice location. The sample size (*n* = 64) was relatively small but adequate for the scope of the study in Martinique. Comparable evaluations of CDSSs in France reported even smaller sample sizes, such as “Dermatoclic” (*n* = 26) (19) a French dermatology CDSS for general practitioners, “Dermatokid” (*n* = 29) (32) for pediatric dermatology and “Antibioclic” (*n* = 32) (33) a tool for antibiotic prescriptions. This study also presents selection biases, inherent to this type of research, with differences from national benchmarks (13) reflecting a selection bias that limits the generalizability of our findings beyond the local context.

Another limitation is the short data collection period (1 month), which may have restricted the number of responses obtained. In addition, the evaluation relied on self-reported data, reflecting physicians’ perceptions and declared use of the tool rather than objective measures of actual use or clinical performance, which is a common limitation of survey-based studies. Our study also did not assess clinical outcomes such as diagnostic accuracy or patient management. In addition, future studies with larger samples should investigate subgroup differences, such as variations in the use of the different sections of the tool according to physicians’ specialty, age, or years of experience, which could provide further insight into usage patterns. A multivariate analysis could also help identify independent predictors of module engagement by accounting for variables such as clinical setting (e.g., hospital vs. private practice), familiarity with digital tools and previous exposure to tutorials related to the tool. Since this study, our tool has progressively expanded in France and other Francophone regions worldwide, now counting more than 5,000 users. This provides an opportunity for larger-scale evaluations to assess its real impact on medical practice, for example by comparing physicians’ responses to clinical cases with and without using our tool, or before and after a defined period of use. We also acknowledge that future third-party validation would be valuable to further strengthen impartiality and objectivity.

### 4.8 Future perspectives or tool improvement

The identification of underused features underscores opportunities for refinement (QR2), while perspectives on AI integration and interoperability highlight the scalability of the tool (QR3).

The tool’s future development could focus on expanding its iconography to cover a wider range of dermatological conditions and refining its diagnostic support by including lesion classification and keyword search functionalities. Additionally, integration with widely used practice management software and interoperability with electronic health records (EHRs) could enhance usability, facilitate daily clinical use, and promote wider dissemination of the tool.

Paid AI tools, such as “VisualDx” (34) and “Dermalyzer” (35), have already demonstrated the potential of machine learning to enhance diagnostic accuracy, underlining the relevance of this direction for our tool. In this context, the integration of artificial intelligence represents a key direction for the future development of the tool. Beyond basic image recognition for lesion classification using deep learning and computer vision techniques, we envision combining this approach with a language model (LLM) capable of asking contextually relevant questions, much like a dermatologist would, to guide general practitioners toward accurate diagnoses and appropriate therapeutic decisions. The development of such an AI agent could build upon our tool existing database of dermatology cases and resources, which offers a robust foundation for training advanced models designed to support clinical decision-making.

Finally, translating the platform into English could significantly broaden its reach and encourage adoption in non-Francophone regions. To be truly useful, it must also adapt to the treatments available locally and reflect regional prescribing habits. Including patient education features could further support safe and effective use in diverse healthcare settings.

In line with the overall research objective (RO), comparative analysis with other CDSS tools highlights our tool’s added value as a scalable and accessible solution for under-resourced settings. Unlike subscription-based international platforms, our tool is freely available, phototype-inclusive, and designed with a modular structure that enhances interoperability.

## 5 Conclusion

In line with our Research Objective (RO), this study evaluated the reliability, usefulness, and effectiveness of a dermatology CDSS in Martinique. The tool was perceived as reliable and relevant, with high usability (SUS 87.7/100) and strong acceptance among physicians, confirming its integration into routine practice (QR1). The limited use of certain modules (Wound Care and Medical Coding) highlights barriers to adoption and provides opportunities for targeted improvements (QR2).

The tool’s adaptability to diverse phototypes and the Martinique context suggests potential scalability to other Francophone or tropical regions, provided that future evaluations confirm its impact on clinical decision-making (QR3). These findings underline both the practical value of our tool for daily dermatological practice and its theoretical contribution as a model of accessible, scalable CDSS. They also set the stage for larger-scale validation studies and international deployment. Future work will include broader cross-regional evaluations and independent validations in other Francophone settings, as well as exploring AI-enhanced functionalities to further support diagnostic accuracy and therapeutic decision-making.

## Data Availability

The raw data supporting the conclusions of this article will be made available by the authors, without undue reservation.
